# Technology-assisted peer therapy: a new way of delivering evidence-based psychological interventions

**DOI:** 10.1186/s12913-022-08233-6

**Published:** 2022-06-30

**Authors:** Najia Atif, Huma Nazir, Zoone Hasan Sultan, Rabia Rauf, Ahmed Waqas, Abid Malik, Siham Sikander, Atif Rahman

**Affiliations:** 1 Human Development Research Foundation, Rawalpindi, Pakistan; 2 Department of Primary Care and Mental Health, Institute of Population Health, University of Liverpool, Liverpool, UK; 3 Health Services Academy, Chak Shahzad, Islamabad, Pakistan; 4 Global Institute of Human Development, Shifa Tameer-E-Millat University, Islamabad, Pakistan

**Keywords:** Perinatal depression, Perinatal mental health, Digital intervention, App for depression, Task-sharing

## Abstract

In low-income settings, ninety percent of individuals with clinical depression have no access to evidence-based psychological interventions. Reasons include lack of funds for specialist services, scarcity of trained mental health professionals, and the stigma attached to mental illness. In recent years there have been many studies demonstrating effective delivery of psychological interventions through a variety of non-specialists. While these interventions are cost-effective and less stigmatising, efforts to scale-up are hampered by issues of quality-control, and what has been described by implementation scientists as ‘voltage-drop’ and ‘programme-drift.’ Using principles of Human Centred Design in a rural setting in Pakistan, we worked with potential users to co-design a Tablet or Smartphone-based App that can assist a lay-person deliver the Thinking Healthy Programme, a World Health Organization-endorsed evidence-based intervention for perinatal depression. The active ingredients of this cognitive-therapy based intervention are delivered by a virtual ‘avatar’ therapist incorporated into the App which is operated by a ‘peer’ (a woman from the neighbourhood with no prior experience of healthcare delivery). Using automated cues from the App, the peer reinforces key therapeutic messages, helps with problem-solving and provides the non-specific but essential therapeutic elements of empathy and support. The peer and App therefore act as co-therapists in delivery of the intervention. The peer can deliver the intervention with good fidelity after brief automated in-built training. This approach has the potential to be applied to other areas of mental health and help bridge the treatment gap, especially in resource-poor settings. This paper describes the process of co-development with end-users and key features of the App.

## Introduction

Depression is the leading cause of disease burden worldwide, affecting over 300 million people globally [1]. The condition prevents individuals from achieving their full potential, drains human capital, and is associated with premature mortality from suicide and other chronic diseases. It represents a major obstacle to achievement of the United Nations’ Sustainable Development Goals, especially in low- and middle-income countries (LMICs) [2]. The treatment gap for psychological interventions, the first-line of management for depression, exceeds 90% in LMICs [3]. Major contributory factors include lack of resources to fund specialist mental health services and the scarcity of trained mental health professionals, in addition to the stigma of seeking care for mental health [4]. However, even in high income countries, the treatment gap exceeds 50% [3]. It is clear that if the challenge of reducing the global burden from depression is to be met, it cannot be business as usual and new strategies will be required.

In recent years several studies, mostly from LMICs, have demonstrated the effective delivery of psychological interventions through a variety of non-specialists [5]. This approach is referred to as ‘Task Shifting (also known as Task Sharing), defined as “delegating tasks to existing or new cadres with either less training or narrowly tailored training” [6]. Task Sharing is advocated as a key strategy to reduce the treatment gap for mental disorders in LMIC by the World Health Organization. One example of this approach is in the area of perinatal depression, a public health priority not only because of the disease burden but also due the effect of maternal depression on mother-infant bonding and childcare and the long-term impact on the infant’s physical and cognitive development [7]. A number of studies have demonstrated that evidence-based psychosocial interventions for perinatal depression can be effectively delivered by non-specialists [5, 8]. In Pakistan, we developed and evaluated the Thinking Healthy Programme (THP) designed to be delivered by community health workers [9–11]. The programme incorporated cognitive behaviour therapy-based techniques focusing on the mother’s mental health and its effect on her relationship with the infant and significant others and inter-session practice activities to help the mother and family to problem-solve [9]. A large randomised controlled trial showed that THP more than halved the rate of depression compared with usual care and led to significant improvements in women’s functioning and disability [10]. In 2015, the Thinking Healthy Programme was incorporated into the World Health Organization’s flagship Mental Health Gap Action Programme (mhGAP) for global dissemination [12]. Studies from South Asia have demonstrated the feasibility of delivering the programme through peers (lay-women from the community with no prior experience of health-care delivery) [13, 14].

However, despite major policy impetus [15], efforts to scale-up the Thinking Health Programme are hampered by issues of quality-control in training and supervision, and what has been described by implementation scientists as ‘voltage-drop’ i.e., the intervention loses some degree of its potency or fidelity when moving from efficacy to effectiveness in the real world, and; ‘programme drift,’ i.e., the intervention deviates from its manualised or implementation protocols [16]. In longer-term follow-up of our randomised trial of peer-delivered Thinking Healthy Programme [17], we found significant reductions in the positive effects of the intervention when compared with the control arm (receiving enhanced usual care), and postulated reasons included the phenomena described above, more likely to occur in interventions delivered by minimally trained non-specialists. Applying a technological solution to this challenge and working with the local community, we aimed to develop an Application (App) which allows non-specialist peers to deliver the intervention sustainably without the need for extensive training and supervision. Our approach ensures the active therapeutic ingredients are delivered to fidelity and at the right dose with the help of the peer-operated App. We envisage this innovation will assist in scale-up of the THP through all types of lay-workers, making the intervention accessible to the most under-served populations. This paper describes the formative research that led to the development of the intervention and its key features.

## Methods

Our aim was to develop a Technology-assisted peer-delivered Thinking Healthy Programme for perinatal depression focusing on the needs of women living in resource-poor rural communities.

### Settings

The study was conducted in rural settings of Rawalpindi District in Punjab, Pakistan. The economy of the rural areas is based largely on subsistence farming, semi-skilled or unskilled labouring or low-paid government service in nearby cities. Each household has on average 6.2 members, and female literacy rate is about 50%. The infant mortality rate is about 84 per 1000 live births. Epidemiological studies from the area indicate high rates of perinatal depression, ranging from 24% to 27% [18, 19]. The study area characterises a typical rural setting in a developing country where basic healthcare is delivered through a network of primary health care centres and community health workers. There is very little provision for screening or treatment for depression or other common mental disorders in these centres, with the nearest public mental health facility located in the city of Rawalpindi about 60 Kms from the study area.

### Design

This usability testing study was approved by the Ethical Review Committees at the Human Development Research Foundation in Pakistan (IRB/0424/2021) and the University of Liverpool in the UK (Reference 10081). All methods were applied in accordance with the Declaration of Helsinki. All participants approached for usability testing provided written informed consent. Participants who were not literate were assisted by the research team who read the informed consent aloud and explained what their participation meant; and further procedures involved in the usability testing such as recording of the interactions with delivery agents. All participants were ensured confidentiality and anonymity and use of the study findings for research purposes only. They were also ensured that they were free withdraw from the study without giving any reason and that their participation is completely voluntary.

We employed principles of Human Centred Design (HCD) to achieve our aim. HCD is an approach to innovation that places the end user at the heart of the process. It hypothesises that only by fully understanding the potential user, their goals, environment, and constraints can we build products and services that optimally address their needs. It has wide applications in global health [20] and psychosocial intervention [21]. As our aim was to develop a digital tool that could assist lay workers in rural Pakistan deliver the Thinking Healthy Programme with good fidelity to women with depression, it was critical that we understood the requirements and preferences of the end-users. It was equally critical to ensure the tool was simple to use and its contents easy to understand, but retained the key ingredients that underpinned the intervention, so that it would not lead to voltage drop or programme drift. The Human Centred Design allowed us to work closely with end users to achieve these aims.

**Fig. 1:**
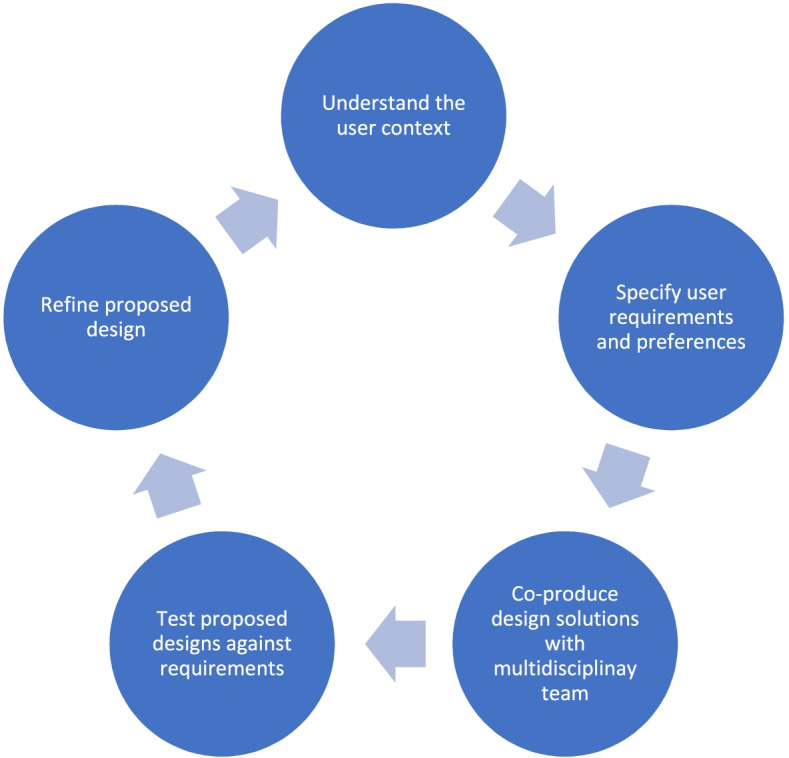
Human-Centred design approach

Key requirements of the HCD approach [22] are: a) Understanding the user context; b) inclusion of multidisciplinary skills and perspectives in the design team; c) user involvement throughout design and development; d) design driven and refined by user-centred evaluation, and; e) an iterative process with frequent feedback loops. These are illustrated in Fig. 1.

We operationalised these key HCD requirements into three iterative steps which were carried out by a specially constituted design team consisting of three distinct groups: a) An 'expert’ group whose members included specialist mental health practitioners (psychologist, psychiatrist) and the developers of THP (*n* = 6); b) A ‘user’ group, selected purposefully and consisting of women who has suffered from perinatal depression (*n* = 4), their husbands (*n* = 2), and community health workers who had experience of delivering THP (*n* = 4), and; c) a ‘technology’ group which had a software developer and a graphic designer. Each step in the design process was undertaken by the three groups working closely and ensuring consensus.

### Step 1: Establish context of use and specify user requirements

In-depth qualitative work to understand the needs of the users in terms of the intervention content and delivery was achieved through our previous work [9, 13]. Our objective here was to analyse user requirements and user preferences related explicitly to the use of technology to assist intervention-delivery. The design team undertook a desk-review and user consultation to develop a Usability Report which captured the profiles, capabilities, requirements, and preferences of end-users. Following this, a detailed power-point storyboard based on the Thinking Healthy Programme manual [12] was developed. It included ideas of avatars to be included in the App, scripts, diagrams, vignettes, screenshots, and interface mock-ups. In this early step of the design process, we pursued many different possible solutions and concepts. The storyboard was continually reviewed by the design team including the user-group at various time-points allowing for rapid turnaround of information related to user requirements. This led to the development of a paper prototype, skeletal framework of technology interface (using tablet or smartphone devices) and mock-up of procedures ready for more detailed testing.

### Step 2: Usability testing with design team

The testable prototype was exposed to controlled tests that considered usability and overall functionality of the prototype. Our testing used the method of ‘cognitive walkthrough’ commonly employed to test web-based interfaces for telemedicine apps and mobile phone apps [22, 23]. In a cognitive walkthrough, the design team members carried out each task required of the App while focusing on cognitive processes that the task required, documenting where they encountered problems. Problems uncovered by the tests were addressed by the design team, with testing repeated if necessary. This led to the development of an advanced prototype.

### Step 3: Usability testing with end-users

All participants approached for usability testing provided written informed consent before participating in the study. An independent team of researchers were trained to conduct the usability testing. Participants who were not literate were assisted by the research team who read the informed consent aloud and explained what their participation meant; and further procedures involved in the usability testing such as recording of the interactions with delivery agents. All participants were ensured confidentiality and anonymity. They were also ensured that they were free to withdraw from the study anytime without giving any reason and that their participation was completely voluntary.

The advanced prototype was exposed to end-users in actual field-setting (such as in their homes where the sessions will be delivered). These end-users were not members of the design team and were purposefully selected to represent the target population in terms of education and social status. Problems uncovered by the tests were addressed by the design team, with the testing cycle repeated if necessary. Test cycles were kept short with a low number of participants (*n* = 6) in each cycle with the same women (*n* = 3) and peer (lay) delivery-agents (*n* = 3) participating in each cycle. User testing involved monitoring users while they interacted with the technology interface. The research team asked the users to think aloud, allowing the observer to gain an insight into the train of thought the user employed as they encountered and attempted to overcome usability and human factors problems. The research team took notes and, where permitted, video or audio recordings.

## Results

For simplicity and to avoid duplication, the feedback of the design team’s user group and the participants of the usability-testing (step 3) are combined and reported in the following sections.

### User profiles and requirements

#### a) Delivery agent

 The App was developed so it could be employed by a lay-therapist in resource-constrained settings. This lay-therapist could be a community health worker, nurse, midwife or even a peer. For design purposes, we profiled a typical peer whom we defined as a “lay woman from the community with shared socio-demographic and life experiences with the target population [24]”. Our previous work showed that meeting a peer to discuss one’s problems was perceived by users to be less stigmatising than seeing a mental health professional in a facility [21]. A typical peer therapist had the following profile: At least 10 years of formal education or the ability to read and comprehend Urdu language; socio-demographic background and life experiences similar to that of the target population; willingness to learn new skills; emotional maturity/range of life experience; good interpersonal skills; ability to relate to mothers and their families; ability to maintain a balance between home and work responsibilities; trustworthy, empathetic, and motivated; some understanding of mother and child health issues through personal experience; fluent in local language; able to move in the community freely.

Our discussions with the user Group revealed that in this rural setting a typical peer with the above profile was likely to possess a mobile phone and to be able to use it independently. However, less than half were likely to possess a smartphone. Only a third of peer households were likely to have a personal computer, laptop, or tablet device. Thus, we designed the App with the needs and requirements of a person with basic reading skills, little or no computer skills, and no experience of the health system or counselling. The protype employed a relatively simple 3 button operation – play, pause and rewind. The programme was off-line and not reliant on an internet connection. However, data could be uploaded off-line and forwarded to a central server when online. Usability testing revealed that peers were able to operate the device successfully after a brief two-hour demonstration by a more experienced peer, even if they were not familiar with technology. The ability to read and comprehend the language of the App was necessary so they could follow written instructions and text embedded within the App.

#### b) Target population

 While the women in the rural setting of this study were similar to their ‘peers’ who would deliver the intervention, there were 3 key differences: First, the women would all have depression which affects their mood, energy levels and concentration. Second, while the peers who deliver the intervention would all have basic literacy skills, about 50% or more women receiving the intervention would be non-literate. Third, only about a third would have independent access to mobile phone and therefore a large majority will be technology-naive.

In order to address effects of both depression as well as low literacy among the participants, simple but effective messages were developed. The content was broken down into small segments (not longer than 3 min) with just one or two key messages in each section. Longer sequences were broken down so and there were regular pauses within segments to allow the peer to interact with the woman, discuss the relevance of that message to her own life situation, and offer encouragement. Our user group emphasised that there should not be an over-reliance on the virtual therapist but a balance between the peer and the technology in terms of time spent in a session. Furthermore, the App did not rely on text but used voice-overs and illustrations, given that many women did not read or write. The illustrations were required to be culturally relevant and acceptable.

The sustained engagement of depressed women with the App-delivered intervention was highlighted as a major challenge. The design team used a *narrative approach* to convey key messages. The approach has great potential for engaging participants, stimulating insight, guiding listeners, challenging deeply embedded beliefs and practices, and facilitating behaviour change [25]. The narratives consisted of brief vignettes (stories) played out by avatars in video-form. The vignettes were developed in consultation with local women who had suffered from depression to depict a variety of everyday challenges and life situations. They aimed to help participants reflect and gain better insight into their own problems, think of alternative perspectives, share personal experiences and suggest ways of dealing with challenges. In addition, these vignettes gently challenged deeply held cultural beliefs and practices, e.g., male gender preference and attitudes towards contraception.

The design team’s user group emphasised the importance of including the husband and other key family members in the intervention. Most men worked long hours and were often not very involved in supporting household responsibilities. It was also felt that in many instances, it would be culturally inappropriate for a man to be present in a session with another woman (peer) in these conservative settings. The user group pointed out that mobile phone ownership was universal amongst men and could be a potential source of engaging the husbands. Voice or text messaging specifically targeting husbands after each session were considered feasible. Majority of the husbands preferred receiving the voice messages over text messages. The message would include the key messages of each session, along with the suggestions of how the husband and other family members could support their wives in accomplishing the targets set for each session.

The user group also pointed out that the intervention should not just be focused on the woman and her husband but include all family members. Health promoting activities involved the whole household and could not be practised in isolation. Family participation was important to make effective use of family members for support and assistance of mother and infant. Shared goals would also help remove the possible ‘paranoia’ of family members, e.g., *‘‘they are brain-washing our womenfolk away from our traditional way of life.’* The content of the intervention was therefore kept as inclusive as possible taking advantage of the fact that the child is perceived to be the common agenda of the entire family, and therefore supporting the mother in child-care activities was a shared responsibility.

### Key features of the Thinking Healthy Programme app

The key features of the protype are summarised in Table 1 and described below.

**Table 1:** Key features of the advanced prototype

Area	Features
General structure	Android Application; eight sessions based on Thinking Healthy Programme manual; Avatar ‘therapist’ and supporting characters; operated by peer; co-designing and user testing to ensure cultural acceptability and feasibility
Active ingredients	Uses CBT-based strategies of cognitive restructuring, behavioural activation and problem solving in 3 key areas: self, relationship with newborn, and relationship with significant others
Fidelity and dose	App-directed session with set time allocated for each segment of therapy; record of goal setting and progress achieved
Training and supervision	In-built training and supervision modules not requiring a specialist trainer; includes step-by-step instructions for operating the App and delivering sessions; in-built peer-assessment of competency; guidelines for peer-supervision
Screening and monitoring	In-built pictorial Community Informant Detection Tool for maternal depression to identify high-risk women; 4-item Patient Health Questions for confirmation and 3-monthly monitoring and risk assessment

#### General structure

**Fig. 2:**
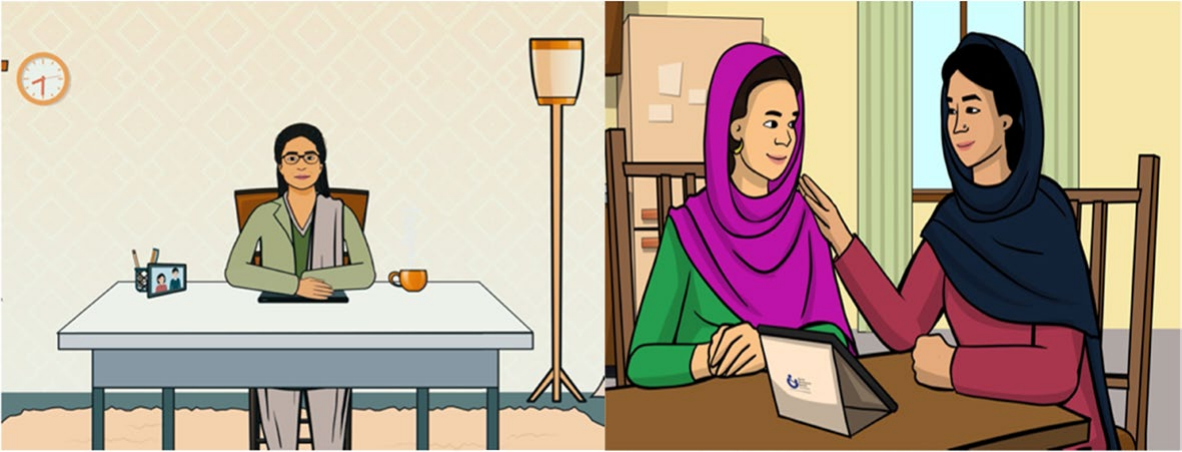
Avatars representing virtual therapist and woman with peer-therapist

All key delivery agent and target population requirements and preferences were incorporated in the final protype which was developed on an Android platform. Eight sessions of the Thinking Healthy Programme training manual [12] were converted into short segments incorporating narrative scripts developed by the Design team. Characters representing a male and a female therapist, women with depression, their family members, and other community members were co-designed with the User group. An artist converted the characters into “Avatars” (graphic image representing each character) (Fig. 2), which were used to voice the narrative scripts.

**Fig. 3:**
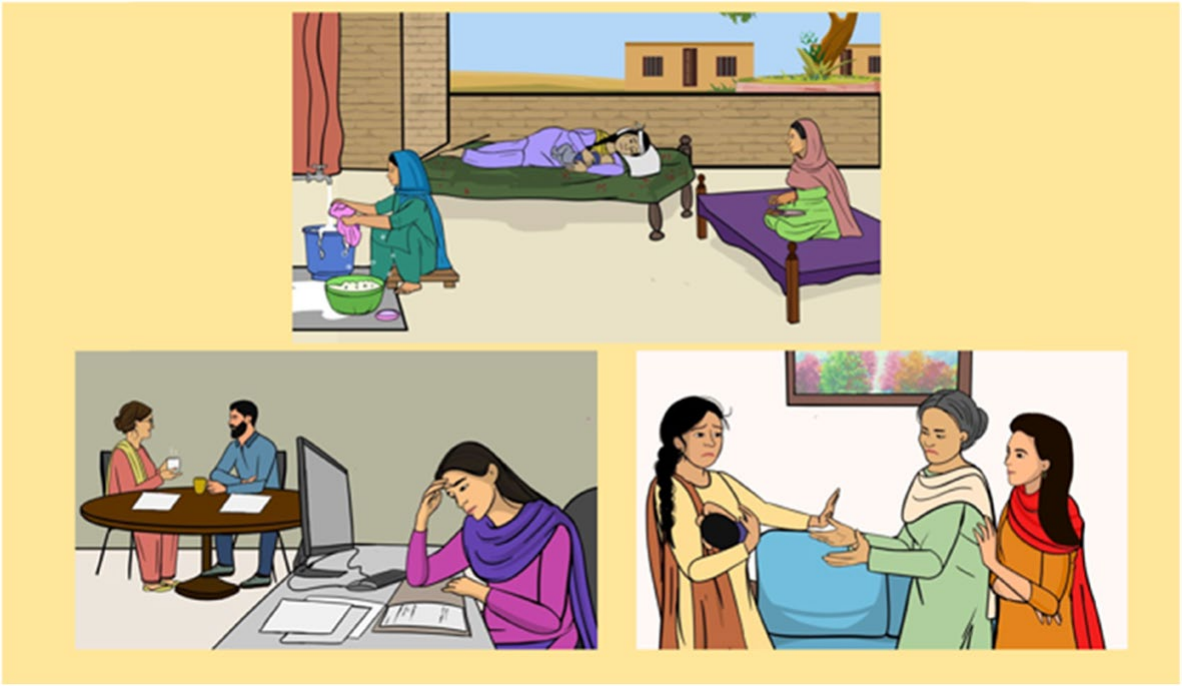
Conveying key messages through a narrative approach

The narratives were interactive, with pause buttons and instructions, allowing the peer and her clients to discuss each scenario in the context of their own lives, agree on goals between sessions, and share problem-solving strategies. The App provides cues and options to the peer to steer the interactions with her clients. The App also employs short, animated stories from lives of women with depression and how they overcame it (Fig. 3). These stories are aimed to provide psychoeducation, engage the client and significant family members with the intervention in a destigmatizing fashion, challenge their unhelpful thoughts and behaviourally activate them. All elements of the App were co-designed and subjected to user-testing in real world settings to ensure cultural acceptability and feasibility.

#### Active ingredients

Previous formative research in similar rural settings indicated that women with perinatal depression had problems in three key areas [9]: a) The woman’s cognitions about her own self – her mood, health and well-being. The depressed women felt helpless and showed a degree of fatalism about their circumstances; they somatised their symptoms and attributed them to superstition; and they considered their health needs secondary to those of their family. b) Thoughts and feelings about the newborn. They worried about their own and infants’ safety during labour and childbirth; felt guilt over lack of positive feeling towards the baby; showed a tendency to blame themselves if the baby was unwell; and worried excessively about the infant’s future. c) Their relationship with family members, friends, and the community. Common themes revolved around feelings of being isolated from and ostracised by the extended family; not living up to ‘expectations’; and feelings of inferiority; lack of confidence and being weighed down by societal codes of conduct.

**Fig. 4:**
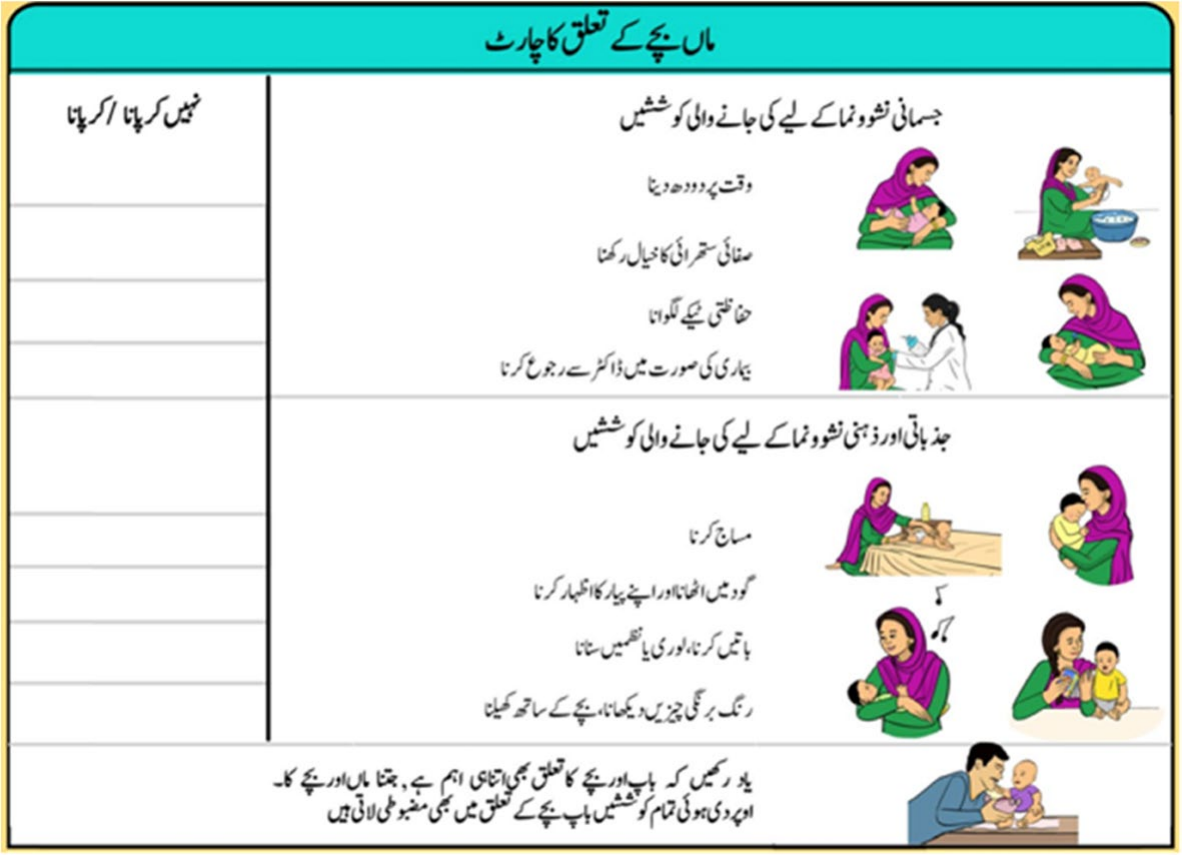
Setting goals to assist with mother-infant bonding

The Thinking Healthy Programme used simplified cognitive behaviour therapy (CBT)-based strategies to address these three broad areas. Key CBT strategies included identification and replacement of unhelpful thoughts and beliefs (cognitive restructuring), behavioural activation and problem solving. These active ingredients were delivered by the virtual ‘avatar’ therapists embedded in the App operated by the peer (Fig. 2). Using automated cues from the App, the peer plays the therapy sections, and introduces pauses to reinforce key therapeutic messages, set goals (Fig. 4), help with problem-solving and provide the non-specific but essential therapeutic elements of empathy and support. Thus, while the App ensures that the above active therapeutic ingredients are delivered to fidelity and at the right dose, our approach does not rely solely on technology, but builds counselling skills of peers to maintain human-contact and empathy.

Fidelity and dosage of intervention: Each therapy session is directed by the App from start to finish. The active ingredients are delivered through the virtual therapist ensuring the appropriate dosage of these key therapeutic elements is maintained in every session.

**Fig. 5:**
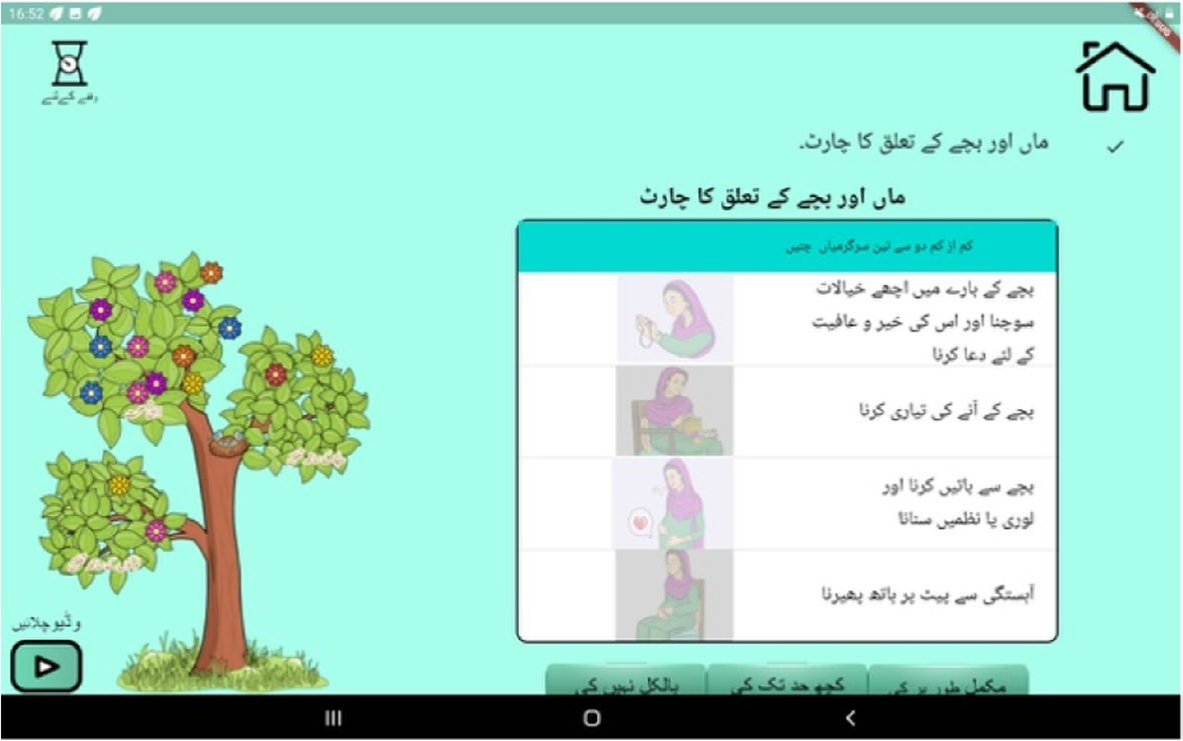
Monitoring progress and problem solving using an interactive 'progress tree' tool

A record of time spent in delivering each individual session is maintained automatically, and this data can be used for monitoring for fidelity. The App also keeps a record of between-session goals set for individual clients. In each session, the peer records the progress achieved in each goal using an interactive ‘progress tree’ which ‘flowers’ as goals are achieved (Fig. 5). Drop down menus with suggestions assist the peer to discuss problem-solving strategies with clients who may be struggling to achieve their goals. A record of goals achieved is automatically recorded in the App. These automated features help ensure each individual session is delivered to fidelity, while the aggregate data from a centre could serve to monitor the quality of programme delivery.

#### Training and supervision

Conventional training of THP is brief (5 days) but conducted by a specialist trainer and relies on experiential learning (i.e., training on-the-job) which requires extensive supervision by the specialist. This poses challenges for scale-up. The App allows non-specialist peers to deliver the intervention sustainably without the need for extensive training and supervision. There is an in-built training module integrated into the App which uses the same ‘avatar’ therapists in ‘trainer’ roles. The first training module focuses on instructions for operating the App and developing the counselling skills of peers, including engagement, empathy, and support. In the second module, the peer learns about each CBT-based active ingredient and its delivery by the ‘avatar’ therapist. It includes session-by-session instructions on how the peer would interact with the client and the App to make the delivery effective. The third module has instructions on dealing with challenging situations, including risk assessment and referral. The training module is best delivered in group settings (preferred), although it can be undertaken individually or in pairs. In group or paired settings, there are in-built practice sessions based on role-plays to consolidate learning. The total training time in group settings is 18 h to allow for role-plays, interactive discussion, and peer-based competency assessment (explained below). The training does not require the need for specialist trainers and can be facilitated by an experienced peer.

Competency in training is assessed through an in-built role-play assessment procedure based on EQUIP [26], an online platform to evaluate common therapeutic factors such as verbal and non-verbal communication skills, demonstration of empathy, collaborative goal setting etc. of non-specialists. Each peer has to perform a role-play with an actor client who is provided with a standard script and rated by the peer trainer (not directly involved in the training), using an in-built rating scale evaluating a number of domains and specific skills to be demonstrated. This evaluation is repeated during peer-supervision.

The App has an in-built supervision module. It is recommended that peers meet in group settings at least once a month for peer-supervision. The App keeps the record of peers’ attendance and duration of the supervision session and collects data on progress of each client, the time taken on each session with an individual client, and any adverse events. It has referral pathways to ensures necessary steps have been taken to address any adverse events. The supervision module allows the peers to discuss progress of individual clients including successes and challenges, and to brainstorm for solutions to problems. The supervision module has links to access the training module for revising the active ingredients of the intervention and to practise them through conducting role plays.

#### Screening and monitoring features

Building on our earlier work [27, 28] we embedded a two-phased identification tool in our App to identify perinatal depression in the community. In Phase 1, the App employs the Community Informant Detection Tool for maternal depression (CIDT-MD) [27] for the identification of high-risk women through key informants in their community. These might include community health workers and other individuals comprising their social network. CIDT-MD consists of pictorial description of key symptoms of perinatal depression linked with non-stigmatizing idioms. This is based on evidence that detection is best achieved by community informants through matching the pictorial description with people the informants encounter routinely in their area of work and responsibility [27]. The illustrations with a brief description can be sent as a single picture to a smartphone or visually demonstrated in-person through the App to a key informant. Phase 1 of screening allows the delivery-agent to identify women who might have or are at increased risk of developing perinatal depression. Phase 2 involves an evaluation of the identified women with the validated 4-item Patient Health Questionnaire (PHQ-4) [28, 29]. The PHQ-4 consists of two items each for depressive symptoms and anxiety symptoms. The depressive symptoms correspond to a sad mood and anhedonia while the anxiety symptoms assessed are nervousness and a lack of control or worrying. This scale was chosen because it is brief, assesses for depression and comorbid anxiety symptoms, and has shown sensitivity and specificity comparable to the original nine-item PHQ-9 [28]. The scale was integrated into the App and administered by peers to women under their care. It was therefore designed to be simple and user-friendly. Each question was accompanied by an illustration and an audio voice-over that could be repeated. The Likert-scale response was converted into a visual scale with touch-sensitive slider scale.

Each woman completes the PHQ-4 at baseline, and then at regular intervals of 3 months. The App provides referral guidelines for women who do not improve, those at risk of harming themselves or others, and those at risk of interpersonal violence. These data are stored in the tablet and transferred to a central server in two ways. First, if the tablet is connected to the Internet, the peer can log on to the server and upload the completed questionnaires. If the peer does not have access to the Internet, she can upload the completed questionnaires when she visits the centre for supervision. The monitoring data serves several purposes. It allows the woman and her family members and peers to visually gauge progress over time. It helps with supervision of the peers, allowing the supervisors to identify problem areas and discuss potential solutions that they can take back to the woman and her family. It assists with decision about referral to specialist services. Finally, the aggregated data allows monitoring of the overall quality of the programme.

## Discussion

Evidence-based psychological interventions for treatable common mental disorders such as depression remain inaccessible to most consumers worldwide. Our innovation of a technology-assisted peer-delivered therapy for perinatal depression, co-designed with users in a low-income setting, has the potential to reduce the treatment gap for this condition and could serve as a model for delivery of psychological interventions for other mental disorders in resource-constrained settings.

The delivery of psychological interventions via digital means, such as on-line and mobile applications (apps), is not new, especially in High Income countries (HICs) [30]. There is evidence for effectiveness of different modes of digital psychotherapy including self-guided treatment, in which consumers use web-based and app-based care to apply therapeutic principles without any additional support; guided digital treatment, in which consumers use digital devices with access to trained support specialists who support and guide their use of the treatment programme; and expert-guided digital treatment, in which consumers use digital devices with help from a professional. Similar tools are increasingly being employed in low- and middle-income countries where the treatment gap is highest [31]. The types of digital interventions are broadly similar to those for HICs. A systematic review and metanalyses of twenty-two randomised controlled trials in LMIC demonstrated moderate improvements in patients with depression or substance abuse receiving digital interventions compared to other interventions. However, two major issues remain unaddressed. First, all reported interventions rely on the internet or mobile phone coverage for online provision of the digital therapy. According to the United Nations, almost half the world’s population, 3.7 billion people, the majority of them women, and most in LMICs, are still offline [32]. An over-reliance on online digital technologies can reinforce and indeed accelerate inequalities by excluding those that remain disconnected. Secondly, a key ingredient and common element of all psychotherapies is the empathy and support that is best possible through direct human contact, ideally with someone from the same sociocultural background and shared life experiences. It is likely to be difficult, and possibly not desirable, to find a digital substitute for direct human empathy and support. This notion is supported by a growing evidence base that many patients with depression prefer in-person, face-to-face interaction to a digital interaction [33, 34].

Our innovation circumvents these two major barriers to digital therapies. It does not rely on the internet or mobile phone service for real-time delivery. The App, once downloaded onto a smartphone or Tablet device, can be operated entirely offline. It requires very basic technology skills from the delivery agent and none from the consumer as it is fully operated by the peer. The target population does not require ownership of a device to obtain the therapy. The peer and App act as co-therapists in delivery of the intervention allowing human contact, empathy, and support. Furthermore, it is a rare example of a digital intervention that has been co-designed with the User community from a low-income setting, incorporating the requirements and preferences of the target population.

Another strength of our innovation is that it is built on the evidence-based Thinking Healthy Programme (THP) [10, 11]. Since its adoption by the World Health Organization’s flagship mental health gap action programme (mhGAP), THP has been tested in a number of countries and regions where it has been shown to be culturally acceptable, feasible and effective over a range of geographical regions and delivery agents including community health workers, nurses and peers [11]. Pooled results of trials from India and Pakistan are especially interesting, demonstrating small effects of peer-delivered THP over enhanced usual care [14], and the long-term retention of peers for intervention delivery [35]. Peers are therefore a potentially abundant human resource to allow equitable access of such interventions to even the most deprived populations. In-built training and supervision features allow scale-up of the intervention without the need for specialist trainers. Our previous work has shown that community health workers and family members can be trained effectively using such digital tools [36].

The key limitation of the study is that it does not investigate the effectiveness of the tech-assisted tool. We therefore do not know if it will prove to be as effective as face-to-face delivery. The next phase of our evaluation involves a randomised controlled trial (RCT) in the study area where the technology-assisted peer-delivered intervention will be compared with conventional face-to-face delivery of the Thinking Healthy Programme. Furthermore, scale-up of the innovation will require collaboration between statutory health providers and non-governmental organizations working for maternal and child health that are likely to employ the innovation. There will be resource implications as peers will require a smartphone or tablet device to train and deliver the intervention and be compensated for their time. Further research into the cost-effectiveness of the innovation is also planned.

Another limitation is that the co-designing took place in one south Asian setting. We intentionally chose a population that was from a low socioeconomic background, non-literate and not digitally connected, thus representing a demography with potentially the greatest degree of inequality. We also chose an intervention that is cross-culturally compatible [11]. However, the App is likely to require some technical and cultural adaptation to make it fit for purpose in diverse sociocultural settings.

An effective tech-assisted peer-delivered intervention will have several implications for policy, practice, and research, especially in LMICs. In Pakistan, the need to scale-up psychological interventions for perinatal depression have been recognised [15] and efforts are underway to achieve this. The COVID pandemic has not only underlined the health system challenges such as overburdened staff and lack of trainers and supervisors, but also highlighted how the digital divide can increase inequalities. Our approach to technology offers directions on how inequalities can be bridged by keeping the most disadvantaged at the heart of the innovation. From a practice perspective, the innovation offers an interesting model of service-delivery, with peers working in partnership with the health system to provide care for depression as the first step in a stepped-care model of care. [37]. With additions, the technology has the potential to assist peers in triaging the target population according to symptom severity and other risks such as suicidality and interpersonal violence at an early stage, allowing for better use of scant specialist resource. The technology also has the potential to provide more personalised therapy by developing algorithms that direct the peer towards automated therapy sessions that are tailored to the needs of individual patients. Analytic methods using machine learning can be employed in future versions of the App to help peers take clinical decisions. Finally, the App has the potential to collect data at a population level about anxiety and depression. This can assist with planning for future services as well as research into the mechanism of action of the intervention in various demographic sub-groups, and implementation outcomes, contributing to reducing the global burden from depression.

## Data Availability

All data generated or analysed during this study are included in this published article.
